# Minimalizing Non-point Source Pollution Using a Cooperative Ion-Selective Electrode System for Estimating Nitrate Nitrogen in Soil

**DOI:** 10.3389/fpls.2021.810214

**Published:** 2022-01-12

**Authors:** Rui Su, Junfeng Wu, Jiandong Hu, Liuzheng Ma, Shakeel Ahmed, Yanyan Zhang, Mukhtar Iderawumi Abdulraheem, Zephania Birech, Linze Li, Can Li, Wentao Wei

**Affiliations:** ^1^Department of Electrical Engineering, Henan Agricultural University, Zhengzhou, China; ^2^Henan International Joint Laboratory of Laser Technology in Agriculture Sciences, Zhengzhou, China; ^3^State Key Laboratory of Wheat and Maize Crop Science, Zhengzhou, China; ^4^Department of Agricultural Science Education, Oyo State College of Education, Lanlate, Nigeria; ^5^Department of Physics, University of Nairobi, Nairobi, Kenya

**Keywords:** nitrate nitrogen, all-solid nitrate ion-selective electrodes, water content calibration function, non-point source pollution, recovery rate

## Abstract

Nitrate nitrogen (${\text{NO}}_{3}^{-}$ -N) in the soil is one of the important nutrients for growing crops. During the period of precipitation or irrigation, an excessive ${\text{NO}}_{3}^{-}$ -N readily causes its leaching or runoff from the soil surface to rivers due to inaccurate fertilization and water management, leading to non-point source pollution. In general, the measurement of the ${\text{NO}}_{3}^{-}$-N relies upon the laboratory-based absorbance, which is often time-consuming, therefore not suitable for the rapid measurements in the field directly. Ion-selective electrodes (ISEs) support the possibility of ${\text{NO}}_{3}^{-}$-N measurement by measuring the nitrate (${\text{NO}}_{3}^{-}$) ions in soil quickly and accurately due to the high water solubility and mobility of ${\text{NO}}_{3}^{-}$ ions. However, such a method suffers from a complicated calibration to remove the influences caused by both temperature and other ions in the measured solution, thus limiting field use. In this study, a kind of all-solid ISE system combined with a temperature sensor and a pH electrode is proposed to automatically measure the concentrations of the ${\text{NO}}_{3}^{-}$-N. In this study, a soil water content calibration function was established, which significantly reduces a relative error (RE) by 13.09%. The experimental results showed that the stabilization time of this electrode system was less than 15 s with a slope of −51.63 mV/decade in the linear range of 10^–5^–10^–2.2^ mol/L. Both the limit of detection of 0.5 ppm of the ${\text{NO}}_{3}^{-}$-N and a relative SD of less than 3% were obtained together with the recovery rate of 90–110%. Compared with the UV-Vis spectroscopy method, a correlation coefficient (*R*^2^) of 0.9952 was obtained. The performances of this all-solid ISE system are satisfied for measuring the ${\text{NO}}_{3}^{-}$-N in the field.

## HIGHLIGHTS

- A cooperative ISEs system is proposed to measure soil nitrate nitrogen.

- The detection limit, RSD and the recovery rate meet practical level.

- Guide inaccurate fertilization and irrigation resulting in non-point source pollution.

- A water content calibration function is applied to improve the measurement precision.

- This ISEs system is validated with samples from the planting zones in central China.

## Introduction

Nitrate nitrogen (${\text{NO}}_{3}^{-}$-N) is an important inorganic nutrient in soil that crops can absorb and use directly (Gebbers and Adamchuk, 2010). The fundamental criterion for determining the nitrogen (N) fertilizer utilization rate during crop growth is confirmed by its concentration in the soil (Burton et al., 2018; Nyameasem et al., 2020). Increasing N fertilizer dosage and application is usually one of the essential ways to boost crop productivity (Van Groenigen et al., 2015; Zhang and Yao, 2017). Both N fertilizer and animal dung contain a significant amount of ${\text{NO}}_{3}^{-}$-N (Pennino et al., 2017). However, an excess of ${\text{NO}}_{3}^{-}$-N is leached from the soil surface and then transported to rivers, lakes, and groundwater during precipitation or irrigation (Sadler et al., 2016; Velusamy et al., 2021). Thus, the improper management of N fertilizer leads to a large amount of ${\text{NO}}_{3}^{-}$-N leaching and causes non-point source pollution (Cui et al., 2020; Mahmud et al., 2020). ${\text{NO}}_{3}^{-}$-N leaching leads to the loss of crop nutrients (Sun et al., 2020). In contrast, it also damages both human and animal health, showing colorectal cancer and Non-Hodgkin lymphoma due to the excessive ${\text{NO}}_{3}^{-}$-N concentration in drinking water (Szpak, 2014; Mary et al., 2018). Therefore, it is important to normalize the measurement of ${\text{NO}}_{3}^{-}$-N in soil (Cho et al., 2018; Richa et al., 2021). Considering the concentrations of ${\text{NO}}_{3}^{-}$-N in soil is readily influenced by environmental factors and changes rapidly, it is necessary to perform *in situ* measurement to evaluate the non-point source pollution (Wang et al., 2015; Ma et al., 2019b).

There are many traditional methods used to measure soil ${\text{NO}}_{3}^{-}$-N, but they are not convenient for use in the field. For example, the UV-Vis spectrophotometry is currently used in laboratories to quantify ${\text{NO}}_{3}^{-}$-N (Burton et al., 2020). Also, the method is professional in operation, and the related chemical reagents bring secondary pollution (Rogovska et al., 2018). Differently, the electrochemical methods exhibit fastness and effectiveness on measuring the concentrations of ${\text{NO}}_{3}^{-}$-N in the soil, which is expected to achieve the *in situ* measurement (Ma et al., 2019a). Adamchuk et al. (2005) created a direct soil testing system that used ISEs to analyze soil nutrients and tracked the spatial variability in nutrient distribution, although the *R*^2^ of ${\text{NO}}_{3}^{-}$-N is only 0.41–0.51 in comparison with laboratory testing. Kim et al. (2007) built a sensor array consisting of N, phosphorus, and potassium-selective electrodes and an argentic/argentic chloride (Ag/AgCl) electrode as reference for the detection of ${\text{NO}}_{3}^{-}$-N in soil with the *R*^2^ value of only 0.89. Moreover, ISEs have also been applied to construct a soil nutrient mapping device, which can generally be mounted on agricultural machinery to map soil conditions over the farm terrain (Sibley et al., 2008). In further measurement of ${\text{NO}}_{3}^{-}$-N, the *R*^2^ value was reached to 0.96 by an improved ISE (Tully and Weil, 2014). Although the abovementioned methods have achieved great progress on ${\text{NO}}_{3}^{-}$-N measurement, there are still many challenges associated with environmental factors and such cannot be used in the field. Temperature, salinity, and soil water content influence should be eliminated to improve precision results. In this study, an automatic cooperative measurement system for detecting the ${\text{NO}}_{3}^{-}$-N in soil was developed. Specifically, this system consists of an all-solid-state ISE, a temperature electrode, and a pH electrode to accurately measure the ${\text{NO}}_{3}^{-}$-N. Moreover, the developed cooperative system is expected to realize the *in situ* measurement of soil ${\text{NO}}_{3}^{-}$-N and providing scientific soil fertilization recommendations at the grassroots level, thus reducing the non-point source pollution. In this system, a peristaltic pump was utilized to control this system working automatically through the microcontroller unit. To lessen the impacts from soil water content, a correction function was introduced to increase the accuracy of on-site measurements toward soil ${\text{NO}}_{3}^{-}$-N. The signals from the cooperative ISE system are transferred to an upper computer in real time.

## Materials and Methods

### Study Area and Soil Sampling

**FIGURE 1:**
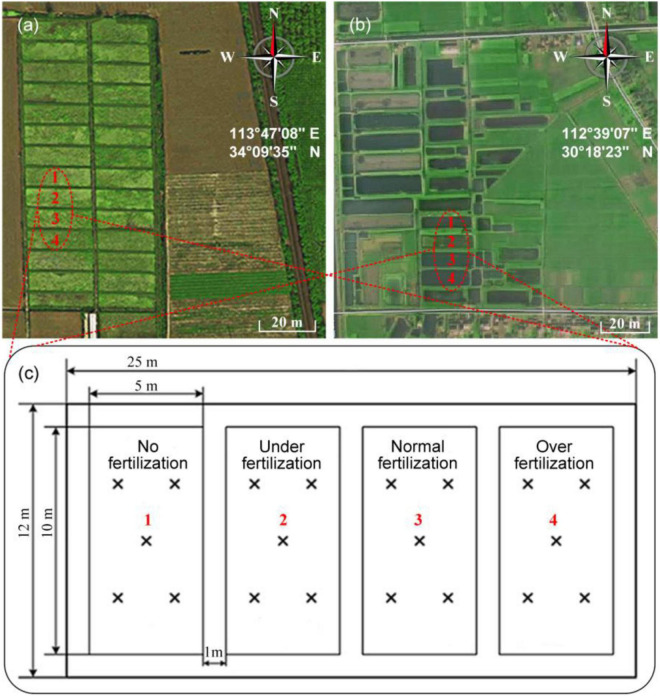
Field experiment. The selected experiment place in **(a)** Xuchang (113°47′08′′E, 34°09′35′′N) and **(b)** Qianjiang (112°39′07′′E, 30°18′23′′N). **(c)** Field fertilization modes in the experiment place and “×” indicates the soil sampling locations.

Field experiments were carried out in the wheat-maize rotation zones, including Xuchang City (113°47′08′′E, 34°09′35′′N), Henan Agricultural University, China (Figure 1a), and the rice growth field in Qianjiang City (112°39′07′′E, 30°18′23′′N), Hubei Province (Figure 1b). In this experiment, the field area of 300 m^2^ was totally divided into four plots. The different fertilization modes were arranged in each plot of 50.0 m^2^ (2.5 m × 20 m). Each plot has six rows of planted crops and a 1-m ridge erected between them. Figure 1c depicts the zones for soil sampling, including (1) control (no fertilization); (2) normal fertilization, i.e., the compound fertilizer of 50 kg/66.7 m^2^ (the urea containing 46% N, calcium superphosphate containing 16% phosphorus pentoxide, and potassium sulfate containing 50% potassium oxide, at a ratio of 14:6:9), (3) under-fertilization (0.5 times), and (4) overfertilization (1.5 times). Other field management practices were identical in each plot. Five soil samples at the depth of 0–20 cm were collected from each plot by the plum blossom pattern method. Notably, 21 soil samples were randomly chosen from two zones, and each soil sample was repeatedly measured six times.

### Cooperative Ion-Selective Electrode System

All-solid-state ISEs (cooperative ISEs) with small volume and fast responses were used to determine the ion concentration (activity) of ${\text{NO}}_{3}^{-}$-N from the membrane potentials in the solution. The dissolved ${\text{NO}}_{3}^{-}$ ions diffuse through the polyvinyl chloride membrane in the ISEs when the electrode was immersed into the solution to test the ${\text{NO}}_{3}^{-}$ ions. When the concentrations on both sides of the membrane reach equilibrium, the membrane potential difference becomes steady. Then, the logarithm of the ${\text{NO}}_{3}^{-}$ concentrations in the external solution is proportional to measured electrode potential, as Eq. 1,


(1) $$\text{E}={\text{E}}^{\text{Θ}}-\frac{2.303\,\text{RT}}{\text{nF}}\,\text{log}\,\left[{\text{NO}}_{3}^{-}\right]$$

Where E is the electrode potential, E^Θ^ is standard apparent electrode potential, including potential difference of membrane/external solution, membrane/internal solution potential, and special interface/internal solution, R is a constant called the universal gas constant (8.314 J K^–1^ mol^–1^), T is the absolute temperature (K), F is the Faraday constant (96,485 C/mol), and *n* = 1 is the charge transfer number for the reduction in ${\text{NO}}_{3}^{-}$.

**FIGURE 2:**
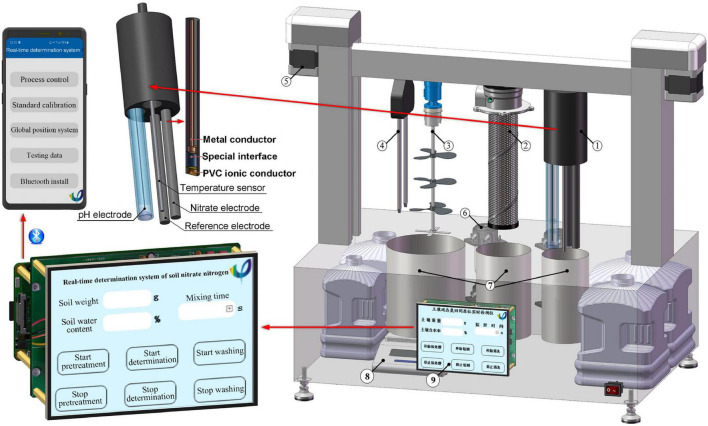
Scheme and profile of the cooperative measurement system: (1) Combination electrode array, (2) Filter, (3) Stirrer, (4) Soil water content sensor, (5) Stepper motor, (6) Peristaltic pump, (7) Sample cell, (8) Electronic scale, and (9) LCD touch screen.

Figure 2 depicts the cooperative ISE system for the determination of the ${\text{NO}}_{3}^{-}$ -N concentrations. The measurement procedure involved: first, placing a soil sample in the pretreatment unit followed by pressing the software button on the LCD touch screen or using the APP in a phone to start the measurement. The soil weight is measured automatically and stored in the microcontroller unit. The peristaltic pump then draws both the deionized water and 2% (V/V) ionic strength adjustment buffer of 2M ammonium sulfate to the sample cell. In the sample cell, a microcontroller-controlled stepper motor alters the height of the water content sensor. Stirring of the sample solution is then achieved *via* an agitator for 3 min. Thereafter, the peristaltic pump draws the supernatant to the detecting unit. In the measurement unit, the ISE system consisting of an ${\text{NO}}_{3}^{-}$-N electrode, a pH electrode, and a temperature sensor then detects the soil ${\text{NO}}_{3}^{-}$-N concentrations, pH, and temperature, respectively. The signal processing circuit transfers the potential difference generated by the electrode array to the microprocessor, and then, the concentration of ${\text{NO}}_{3}^{-}$-N, which is calculated after the compensation, has been done with soil water content. The collected data are then stored in the microcontroller unit and transmitted to the APP in a phone through the Raspberry Pi’s Bluetooth interface. Once the measurement is completed, the peristaltic pump launches again to automatically clean these electrodes with deionized water.

### *Ab initio* Calculation

**FIGURE 3:**
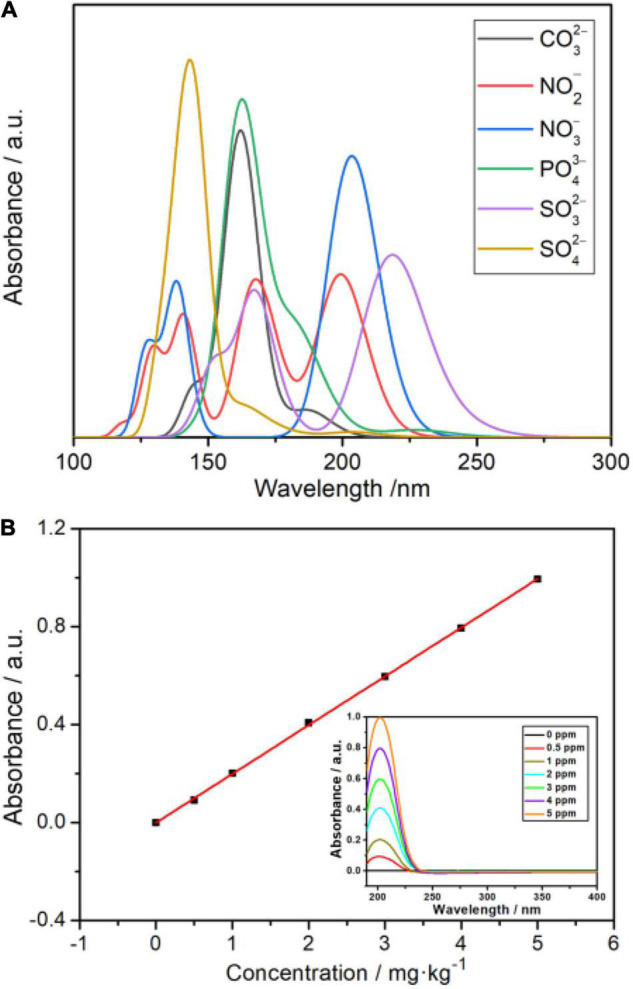
**(A)** Absorbance of several ions in water from UV-Vis spectrophotometry. **(B)** Standard curves were made by UV-Vis spectrophotometry.

The geometries of ${\text{NO}}_{2}^{-}$, ${\text{NO}}_{3}^{-}$, and ${\text{SO}}_{4}^{2-}$ were optimized at the M06-2X/6-311+G(2d,p) level of theory. The frequency calculation was performed at the same level of theory to ensure that the optimized configurations are located at the minimum of the potential surface. According to the benchmark of Zhao and Truhlar (2007), the UV-Vis spectrophotometer was calculated at the M06-2X/6-311+G(2d,p) level of theory with the assistance of time-dependent density functional theory (Li et al., 2020), and the solvation model density (Marenich et al., 2009) was used to represent the water solution situation. All the above *ab initio* calculations were performed by ORCA 5.0.1 programmer (Neese, 2011). The UV-Vis spectrophotometer (Feller Instrument limited business, Nanjing) was used to check the absorption peak positions of each interfering ion. The site of the maximum absorption peak of ${\text{NO}}_{3}^{-}$ was obviously identified to be at a wavelength of 203 nm (Figure 3A). Thus, the standard curves for the UV-Vis spectra method can be obtained (Figure 3B). To confirm the maximum absorption peak of ${\text{NO}}_{3}^{-}$ in UV-Vis absorbance spectra, the *ab initio* calculation is accurately performed first. Later, we can manage these experiments to establish the standard curve at the maximum absorption peak of ${\text{NO}}_{3}^{-}$ The value of the maximum absorption peak seriously influences the correlation coefficient (*R*^2^) obtained from both the ISE system and UV-Vis absorbance spectra.

## Results and Discussion

### Water Content Calibration Function

**FIGURE 4:**
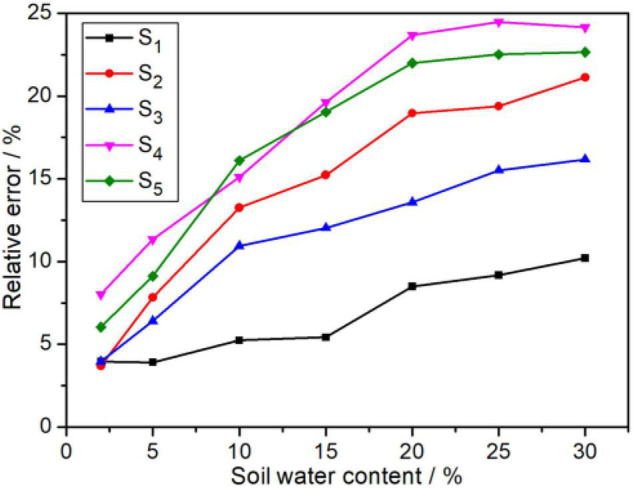
Effects of soil water content on detection accuracy.

Normally, the soil water contents in the field change from 5 to 20%. The soil with a water content of less than 5% is too dry for crop growth, and ${\text{NO}}_{3}^{-}$-N is easily leached out away when the water content exceeds 25%. Notably, 35 measured samples with various water content (2, 5, 10, 15, 20, 25, and 30%) were obtained by adding deionized water into the five dried soil samples (marked as S_1_, S_2_, S_3_, S_4_, and S_5_). The influence of soil water content on the ${\text{NO}}_{3}^{-}$-N concentration measured from this ISE system is shown in Figure 4. Obviously, the influence of soil water content on ISE determination results cannot be ignored. It was observed that the relative error (RE) of ${\text{NO}}_{3}^{-}$-N grew up to 25% rapidly when the water content increases. Even if the soil water content is less than 2%, the RE is found to be greater than 3%. In addition, the RE toward soil water content is a slight difference, which may be caused by their distinguishing intrinsic properties.

As a result, it is necessary to provide a calibration function to remove the impact of inaccuracy caused by soil water content on concentration measurement uncertainty. The relationship is expressed as follows:


(2) $$C_{W}=\left[\frac{C}{m}\times \left(1+\frac{\omega }{100}\right)+\frac{\omega }{\rho_{\omega }\times 100}\right]\times \beta$$

Where *C*_*W*_ denotes the calibrated ${\text{NO}}_{3}^{-}$-N concentration (mg/kg), *C* denotes the volume of extraction solution (ml), *m* denotes the weight of the fresh soil sample (g), ω represents the soil water content (%),ρ_ω_ represents the density of water at the room temperature (1.00 g/cm^3^), and β denotes the mass fraction of soil ${\text{NO}}_{3}^{-}$-N that is calculated from the measured curve (mg/kg).

Table 1 displays the results from 10 soil samples with water contents ranging from 0 to 30%. The results of the ISE system were compared with the UV-Vis spectrophotometry. The RE was fluctuated up to −24.02% before the measurement findings were calibrated by the calibration function. However, after the water content function was added, it dropped to −10.93%. The RE was effectively reduced to 13.09%.

**TABLE 1:** ${\text{NO}}_{3}^{-}$-N concentrations in soil with different soil water contents.

Sample No.	Water content/%	${\text{NO}}_{3}^{-}$-N ISE system/(mg/kg)		${\text{NO}}_{3}^{-}$-N UV-Vis spectrophotometry/(mg/kg)	RE/%	
		Uncalibrated	Calibrated		Uncalibrated	Calibrated
1	0	45.37	45.37	49.35	−8.07	−8.07
2	3.72	50.59	52.85	52.24	−3.16	1.17
3	6.82	29.24	31.63	33.83	−13.59	−6.51
4	8.26	16.45	18.08	19.30	−14.77	−6.32
5	12.88	12.40	14.32	13.55	−8.50	5.64
6	16.62	11.05	13.26	12.82	−13.78	3.42
7	18.67	27.28	33.39	30.86	−11.60	8.20
8	22.91	6.55	7.68	8.62	−24.02	−10.93
9	27.38	30.65	40.72	37.11	−17.39	9.75
10	30.32	40.35	55.03	49.94	−19.21	10.19

### Performance Analysis

**FIGURE 5:**
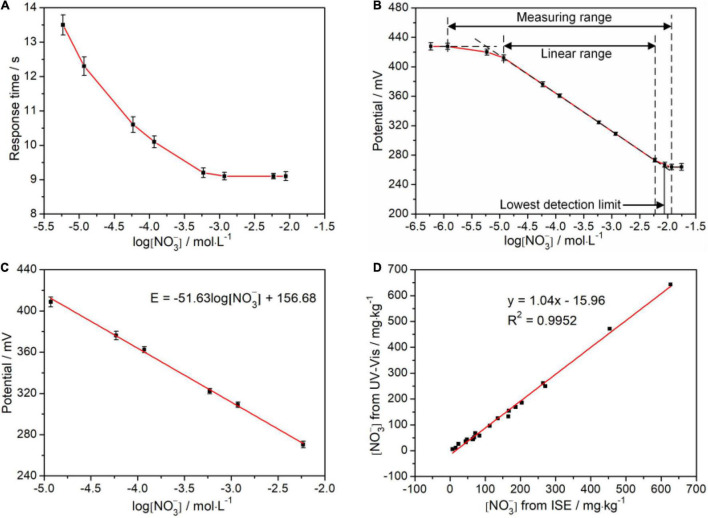
Validation analyses of the ion-selective electrode (ISE) system. **(A)** Response time; **(B)** Linear range; **(C)** Response potential; and **(D)** Correlation between the results from ISE system and UV-Vis spectrophotometry. The (${\text{NO}}_{3}^{-}$) used in **(A–C)** is derived from standard solution. The (${\text{NO}}_{3}^{-}$-N) used in **(D)** is derived from the soil sample solution.

To evaluate this cooperative all-solid-state ISE system, the response time, which is defined as the time interval of electrode potential reaching 95% of the initial potential, was investigated. Figure 5A illustrates the change of the response time at the various logarithm of (${\text{NO}}_{3}^{-}$). It can be observed that the steady potentials can be obtained within less than 15 s, which is comparable with the previous report. Notably, the response time shortens with increasing the (${\text{NO}}_{3}^{-}$), implying that the higher (${\text{NO}}_{3}^{-}$) accelerates the diffusion from the external solution to the internal solution driven by concentration difference and makes the electrode potential steady more quickly. In addition, we studied the linear response interval of electrode potential toward the logarithm of (${\text{NO}}_{3}^{-}$). As shown in Figure 5B, the linear range of (${\text{NO}}_{3}^{-}$) can be directly determined as 10^–5^–10^–2.2^ mol/L. Subsequently, we further studied the rationality of the measured electrode potential in the linear range *via* linear fitting by the Nernst equation of Eq. 2, as shown in Figure 5C. The slope is −51.63 mV/decade which is laid in the rational range of (−54 ± 5) mV/decade. The vertical intercept is 156.68 mV, which is related to E_0_, pH, and T. Notably, recovery rates were also analyzed on all standard samples used in the above experiments, and the results were in an acceptable range of 90–110% (Table 2).

**TABLE 2:** Recovery rate of the ion-selective electrode (ISE) system on detecting ${\text{NO}}_{3}^{-}$-N.

Initial sample concentration^α^ mg/kg	Added standard solution concentration^γ^ mg/kg	Total concentration after adding standard solution mg/kg	Recovery rate/(%)
5	3	3.90	93.33%
5	10	7.96	109.20%
25	15	20.59	107.87%
25	50	36.72	96.88%
50	100	76.95	103.90%
100	200	147.64	97.64%
200	400	305.81	102.91%

*^α^The sample volume is 20 ml. ^γ^The added standard solution volume is 20 ml.*

We explored the reliability of the cooperative all-solid-state ISE system toward UV-Vis spectrophotometer with an *R*^2^ of 0.9952, as shown in Figure 5D. The fitted slope is 1.04, indicating the measured results from our ISE system almost reach to that of the UV-Vis spectrophotometer. Nevertheless, the fitted vertical intercept is −15.96 mg/kg, suggesting the existence of hysteresis effect resulting from the residual (${\text{NO}}_{3}^{-}$) in each measurement. Each measurement was repeated six times with both methods, respectively. The relative SDs (RSDs) were calculated to verify the precision. From Table 3, the RSD of the cooperative ISE system is within 3%, higher than 1% of the UV-Vis spectrophotometry. In comparison with the non-contacting UV-Vis method, the contacting measurement of the ISE system inevitably causes liquid junction, wetting, and clean problem, which leads to the fluctuation of measured potentials and increases the RSD. Thus, such measurement repeatability of our ISE system is acceptable for use in the field. To further verify whether there is a significant difference in the precision of both methods, the *F*-test was conducted in ANOVA. As shown in Table 4, there was no statistically significant difference (*P* < 0.05) between the all-solid-state ISE system and UV-Vis spectrophotometry. This proves that the electrode system can achieve comparable and relatively accurate results that meet the requirements of ${\text{NO}}_{3}^{-}$-N measurement in the field.

**TABLE 3:** Contrast analysis between the cooperative ISE system and UV-Vis spectrophotometry.

Sample No.	Cooperative ISE system		UV-Vis spectrophotometry	
	
	${\text{NO}}_{3}^{-}$-N mg/kg	RSD (%)	${\text{NO}}_{3}^{-}$-N mg/kg	RSD (%)
1	166.97	2.06	154.32	1.02
2	186.53	1.94	169.16	0.77
3	165.56	2.08	132.72	1.03
4	264.25	1.77	261.20	0.49
5	112.80	1.60	96.50	0.71
6	15.88	3.79	11.19	1.40
7	23.64	3.93	26.53	0.78
8	6.83	3.96	5.84	0.15
9	48.15	2.87	43.10	0.77
10	83.68	2.03	58.39	1.05
11	44.23	2.71	35.76	1.54
12	68.48	2.47	51.78	0.58
13	71.59	2.39	67.77	1.52
14	65.52	2.82	44.52	0.77
15	64.10	2.20	44.10	0.61
16	45.19	2.85	33.13	0.91
17	135.45	1.46	124.86	0.34
18	203.61	2.17	185.53	0.66
19	270.89	1.81	249.71	0.61
20	626.64	0.78	643.20	0.32
21	453.72	1.19	471.55	0.59
Average		2.33		0.79

**TABLE 4:** *F*-test of significance in the regression analysis of the experiment results.

Project	Statistic	Project	Statistic	Project	Statistic
Regression equation	*y* = 1.039*x*−15.956	Coefficient *a* test	*a* = 1.039	Intercept *b* test	*b* = −15.956
SSR	509777.523 v = 1	*t*-value (a)	62.713	*t*-value (b)	−4.560
SSE	2462.746 v = 19	*P*-value (a)	0.000	*P*-value (b)	0.000
*F*-value	3932.916	S(a)	0.017	S(b)	3.500
*P*-value	0.000	95% CI	1.004∼1.074	95% CI	−23.281∼−8.632
Test result	*P* < 0.05, refuse H0, accept H1, indicating that there is a significant linear relationship between the two detection methods			*P* > 0.05, accept H0, indicating that there is no significant difference between intercept and 0 value	
					

Furthermore, compared with other ISE detection methods previously reported (Table 5), the detection of ${\text{NO}}_{3}^{-}$-N using ISEs had a wider linear range and a relatively low limit of detection (LOD), the linear range was wider than that of UV-Vis spectrophotometry detection of ${\text{NO}}_{3}^{-}$-N, and the detection speed was faster. The detection method is simple to operate and does not require the participation of professionals, which is providing rational suggestions for relieving the leaching of ${\text{NO}}_{3}^{-}$-N during fertilization and irrigation.

**TABLE 5:** Performance comparison on ${\text{NO}}_{3}^{-}$-N measurement with different techniques.

Detection methods and principle	Measurement ranges	Sensitivity/response times	Limit of detection (LOD)	*R* ^2^	References
DSM^a^	−	−58.2 mV/decade	−	0.41−0.51	Adamchuk et al., 2005
ISE (PVC+Hitachi)	0.11−109.8 mg/L	−	−	0.89	Kim et al., 2007
SNMS^b^	−	6 s	−	0.93	Sibley et al., 2008
ISE (Horiba B-342)	6.8−68 mg/L	−	2 mg/L	0.96	Tully and Weil, 2014
Cooperative ISEs	10^–5^−10^–2.2^ mol/L	<15 s	10^–5.23^ mol/L	0.99	This work

*^a^A method of direct soil measurement (DSM) using ISEs.*

*^b^Soil nitrate mapping system.*

## Conclusion

To detect ${\text{NO}}_{3}^{-}$-N in the field, an electrode system consisting of all-solid-state ${\text{NO}}_{3}^{-}$ ISEs, temperature electrode, and pH electrode was built. A microprocessor-controlled peristaltic pump extracted the measured sample solution automatically in the sample cell. The ${\text{NO}}_{3}^{-}$-N concentration measured by the ISE system was quantitatively calibrated by adding soil water content calibration formula. The RE is effectively reduced to 13.09%. Four different fertilization treatments were carried out in experimental fields of crop growing areas in Henan and Hubei provinces, each area used a plum-shaped cloth point approach, and the cloth point was measured using the ISE system. In the measurement range of 10^–5^–10^–2.2^ mol/L, the ISE system has a response time of less than 15 s with a slope laid in the rational range of (−54 ± 5) mV/decade. The recovery rate of 90–110% has been confirmed from the ISE system for soil ${\text{NO}}_{3}^{-}$-N. Both the RSDs of 3% of the soil ${\text{NO}}_{3}^{-}$-N were obtained from this ISE system. Compared to the classical UV-Vis spectrophotometer, an *R*^2^ of 0.9952 has been obtained. The linear regression *F*-test has been carried out, and there was a significant linear relationship between the measurement results of the two detection systems. We believe that this system can be further optimized and generalized in agriculture, providing rational suggestions for relieving the leaching of ${\text{NO}}_{3}^{-}$-N during fertilization and irrigation.

## Data Availability Statement

The original contributions presented in the study are included in the article/Supplementary Material, further inquiries can be directed to the corresponding author.

## Author Contributions

RS: conceptualization, writing-original draft, and writing-review and editing. JW: validation and writing-review and editing. JH: conceptualization, validation, and writing-review and editing. LM: formal analysis and investigation. SA: writing-review and editing. YZ: formal analysis and supervision. MA and ZB: supervision. LL and WW: software and formal analysis. CL: data curation and project administration. All authors contributed to the article and approved the submitted version.

## Conflict of Interest

The authors declare that the research was conducted in the absence of any commercial or financial relationships that could be construed as a potential conflict of interest.

## Publisher’s Note

All claims expressed in this article are solely those of the authors and do not necessarily represent those of their affiliated organizations, or those of the publisher, the editors and the reviewers. Any product that may be evaluated in this article, or claim that may be made by its manufacturer, is not guaranteed or endorsed by the publisher.

## References

[B1] Adamchuk V. I. Lund E. D. Sethuramasamyraja B. Morgan M. T. Dobermann A. Marx D. B. (2005). Direct measurement of soil chemical properties on-the-go using ion-selective electrodes. *Comput. Electron. Agric.* 48 272–294. 10.1016/j.compag.2005.05.001

[B2] Burton L. Dave N. Fernandez R. E. Jayachandran K. Bhansali S. (2018). Smart gardening IoT soil sheets for real-time nutrient analysis. *J. Electrochem. Soc.* 165 B3157–B3162. 10.1149/2.0201808jes

[B3] Burton L. Jayachandran K. Bhansali S. (2020). Review—the “Real-Time” revolution for in situ soil nutrient sensing. *J. Electrochem. Soc.* 167:037569. 10.1149/1945-7111/ab6f5d

[B4] Cho W.-J. Kim H.-J. Jung D.-H. Kim D.-W. Ahn T. I. Son J.-E. (2018). On-site ion monitoring system for precision hydroponic nutrient management. *Comput. Electron. Agric.* 146 51–58. 10.1016/j.compag.2018.01.019

[B5] Cui M. Zeng L. Qin W. Feng J. (2020). Measures for reducing nitrate leaching in orchards:a review. *Environ. Pollut.* 263:114553. 10.1016/j.envpol.2020.114553 32311625

[B6] Gebbers R. Adamchuk V. I. (2010). Precision agriculture and food security. *Science* 327 828–831. 10.1126/science.1183899 20150492

[B7] Kim H.-J. Hummel J. W. Sudduth K. A. Motavalli P. P. (2007). Simultaneous analysis of soil macronutrients using ion-selective electrodes. *Soil Sci. Soc. Am. J.* 71 1867–1877. 10.2136/sssaj2007.0002

[B8] Li X. Govind N. Isborn C. DePrince A. E. III Lopata K. (2020). Real-time time-dependent electronic structure theory. *Chem. Rev.* 120 9951–9993. 10.1021/acs.chemrev.0c00223 32813506

[B9] Ma L. Li Z. Birech Z. Li S. Yang Y. Zhang W. (2019b). Multi-channel optoelectronic measurement system for soil nutrients analysis. *Electronics* 8:451. 10.3390/electronics8040451

[B10] Ma L. Duan T. Hu J. (2019a). Application of a universal soil extractant for determining the available NPK: a case study of crop planting zones in central China. *Sci. Total Environ.* 704:135253. 10.1016/j.scitotenv.2019.135253 31818585

[B11] Mahmud M. Ejeian F. Azadi S. Myers M. Asadnia M. (2020). Recent progress in sensing nitrate, nitrite, phosphate, and ammonium in aquatic environment. *Chemosphere* 259:127492. 10.1016/j.chemosphere.2020.127492

[B12] Marenich A. V. Cramer C. J. Truhlar D. G. (2009). Universal solvation model based on solute electron density and on a continuum model of the solvent defined by the bulk dielectric constant and atomic surface tensions. *J. Phys. Chem. B.* 113 6378–6396. 10.1021/jp810292n 19366259

[B13] Mary W. Rena J. Jean B. Theo D. K. Peter W. Bernard N. (2018). Drinking water nitrate and human health: an updated review. *Int. J. Environ. Res. Public Health* 15:1557. 10.3390/ijerph15071557 30041450 PMC6068531

[B14] Neese F. (2011). The ORCA program system. *WIRES Comput. Mol. Sci.* 2 73–78. 10.1002/wcms.81

[B15] Nyameasem J. K. Reinsch T. Taube F. Domozoro C. Y. F. Marfo-Ahenkora E. Emadodin I. (2020). Nitrogen availability determines the long-term impact of land use change on soil carbon stocks in grasslands of southern Ghana. *SOIL* 6 523–539. 10.5194/soil-6-523-2020

[B16] Pennino M. J. Compton J. E. Leibowitz S. G. (2017). Trends in drinking water nitrate violations across the United States. *Environ. Sci. Technol.* 51 13450–13460. 10.1021/acs.est.7b04269 29052975 PMC5764095

[B17] Richa A. Fizir M. Touil S. (2021). Advanced monitoring of hydroponic solutions using ion-selective electrodes and the internet of things: a review. *Environ. Chem. Lett.* 19 3445–3463. 10.1007/s10311-021-01233-8

[B18] Rogovska N. Laird D. A. Chiou C.-P. Bond L. J. (2018). Development of field mobile soil nitrate sensor technology to facilitate precision fertilizer management. *Precis. Agric.* 20 40–55. 10.1007/s11119-018-9579-0

[B19] Sadler R. Maetam B. Edokpolo B. Connell D. Yu J. Stewart D. (2016). Health risk assessment for exposure to nitrate in drinking water from village wells in Semarang, Indonesia. *Environ. Pollut.* 216 738–745. 10.1016/j.envpol.2016.06.041 27400904

[B20] Sibley K. J. Astatkie T. Brewster G. Struik P. C. Adsett J. F. Pruski K. (2008). Field-scale validation of an automated soil nitrate extraction and measurement system. *Precis. Agric.* 10 162–174. 10.1007/s11119-008-9081-1

[B21] Sun J. Li W. Li C. Chang W. Peng M. (2020). Effect of different rates of nitrogen fertilization on crop yield, soil properties and leaf physiological attributes in banana under subtropical regions of China. *Front. Plant Sci.* 11:613760. 10.3389/fpls.2020.613760 33408734 PMC7779679

[B22] Szpak P. (2014). Complexities of nitrogen isotope biogeochemistry in plant-soil systems: implications for the study of ancient agricultural and animal management practices. *Front. Plant Sci.* 5:288. 10.3389/fpls.2014.00288 25002865 PMC4066317

[B23] Tully K. L. Weil R. (2014). Ion-selective electrode offers accurate, inexpensive method for analyzing soil solution nitrate in remote regions. *Commun. Soil Sci. Plant Anal.* 45 1974–1980. 10.1080/00103624.2014.912297

[B24] Van Groenigen J. W. Huygens D. Boeckx P. Kuyper T. W. Lubbers I. M. Rütting T. (2015). The soil N cycle: new insights and key challenges. *SOIL* 1 235–256. 10.5194/soil-1-235-2015

[B25] Velusamy K. Periyasamy S. Kumar P. S. Vo D. Sindhu J. Sneka D. (2021). Advanced techniques to remove phosphates and nitrates from waters: a review. *Environ. Chem. Lett.* 19 3165–3180. 10.1007/s10311-021-01239-2

[B26] Wang J. Lü G. Guo X. Wang Y. Ding S. Wang D. (2015). Conservation tillage and optimized fertilization reduce winter runoff losses of nitrogen and phosphorus from farmland in the Chaohu Lake region, China. *Nutr. Cycl. Agroecosyst.* 101 93–106. 10.1007/s10705-014-9664-3

[B27] Zhang H. Yao Y. (2017). Vermiculite addition to soil decreases N water pollution by over 30%. *Environ. Chem. Lett.* 15 507–513. 10.1007/s10311-017-0631-5

[B28] Zhao Y. Truhlar D. G. (2007). The M06 suite of density functionals for main group thermochemistry, thermochemical kinetics, noncovalent interactions, excited states, and transition elements: two new functionals and systematic testing of four M06-class functionals and 12 other functionals. *Theor. Chem. Acc.* 120 215–241. 10.1007/s00214-007-0310-x

