# Supplementary figures and images for: Minimalizing Non-point Source Pollution Using a Cooperative Ion-Selective Electrode System for Estimating Nitrate Nitrogen in Soil
Source: Front Plant Sci. 2022 Jan 12;12:810214. doi: 10.3389/fpls.2021.810214 (PMC8790048; doi:10.3389/fpls.2021.810214)

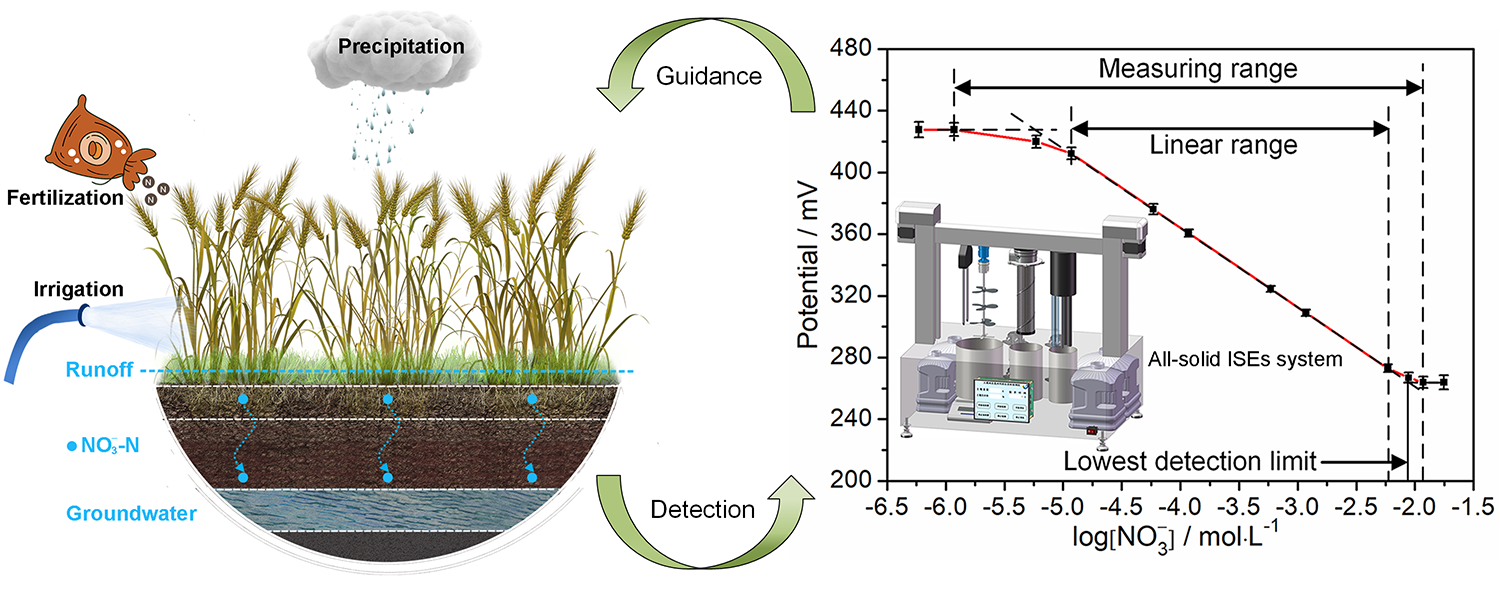

Supplement: Supplementary file 1 [file Image_1.TIF]
